# Exploring attitudes towards seeking help for mental health problems among university students from racially minoritised backgrounds: a systematic review and thematic synthesis

**DOI:** 10.1186/s12889-025-22521-w

**Published:** 2025-04-16

**Authors:** Rosa Hardy, Helen West, Peter Fisher

**Affiliations:** 1 Primary Care and Mental Health, Institute of Population Health, University of Liverpool, Eleanor Rathbone Building, The University of Liverpool, 74 Bedford Street South, Liverpool, L69 7ZA UK; 2 Department of Psychology, Institution of Population Health, University of Liverpool, Eleanor Rathbone Building, The University of Liverpool, 74 Bedford Street South, Liverpool, L69 7ZA UK

**Keywords:** University students, Racially minoritised, Mental health, Attitudes, Help-seeking

## Abstract

**Background:**

University students from racially minoritised backgrounds are at an increased risk of experiencing mental health difficulties but are less likely to seek support compared to students from racial and ethnic majority backgrounds. To increase the accessibility and appropriateness of mental health support for university students, it is important to understand the attitudes towards seeking help for mental health of underserved student groups. This is the first systematic review to synthesise the available qualitative data which explores attitudes toward seeking help for mental health problems among students from racially minoritised backgrounds.

**Methods:**

This systematic review includes qualitative studies exploring attitudes towards seeking help for mental health difficulties among racially minoritised university students. A literature search was carried out using PsycINFO, CINAHL, Medline and Web of Science in March 2024. Participants were racially minoritised university students. Data were synthesised using a thematic synthesis.

**Results:**

Of 493 papers identified, 15 were included in the final thematic synthesis following methodological appraisal of their quality using the Critical Appraisal Skills Programme. There were a total of 314 participants across all included papers. Four analytical themes were identified: “cultural attitudes” outlined how culturally specific experiences of stigma, lack of conversations about mental health, faith, and gender influenced attitudes; “interpersonal relationships” explored the impact of family and peer relationships on attitudes; “psychological barriers” described how psychological constructs, such as preference for self-reliance and feared consequences of disclosure, were culturally-informed barriers to help-seeking; and “systemic barriers” encompassed the structural barriers, discriminatory practices and perceived cultural incompetence of services and institutions that negatively impacted on attitudes towards help-seeking for mental health difficulties.

**Conclusion:**

Culture, identity and social inequality inform attitudes towards help-seeking among racially minoritised students. Exploration of how these factors interact with university systems may improve the provision of mental health support. Systemic change is needed within universities and mental health services to tackle inequality and improve support for racially minoritised students.

## Introduction

There are an estimated 254 million students enrolled at universities globally [1]. Universities worldwide have undergone rapid and diverse changes, such as greater accessibility of higher education for a more diverse student group. Currently, 25% of domicile UK university students are from racially minoritised backgrounds [2]. This does not include the approximately 600,000 international students currently studying in the UK, many of whom will also be racially minoritised [2]. In the US, the percentage of students from Latinx, Black and Asian backgrounds has increased from 15.36% in 1976 to 45.23% in 2022 [3, 4]. Globally, many Higher Education Institutes (HEIs) have had to quickly adapt to the needs of their students. Universities have a duty of care to both protect the human rights of students and staff, and their physical and mental wellbeing [5].

A key aspect of adapting to the changing student population has been developing mental health support services. In 2022, 57% of UK university students reported experiencing a mental health difficulty [6]. In the US, a longitudinal study across 196 universities found that suicidal ideation increased by approximately 90%, from 5.8% in 2007 to 10.8%, in 2016–2017 [7]. University students from racially minoritised backgrounds are at an increased risk of mental health issues [8–11]. In the US, students of colour are more likely to experience symptoms of clinical depression than students from other racial/ethnic groups [3, 12]. Furthermore, multiracial students in the US are more likely to experience suicidal ideation and to have attempted suicide than White students [13]. It is important that universities understand the mental health needs and attitudes towards seeking help of students. Identifying underserved student populations and exploring why they are reticent to seek support is essential not only to increase their engagement with mental health support but also to enable universities to create a more inclusive and supportive environment that better meets the needs of their diverse student groups [8, 10–12].

### The psychological impact of discrimination and racism on mental health

The minority stress model proposes that discrimination and stigma experienced by minoritised individuals contributes to adverse health outcomes [14]. Furthermore, minority status leads to exposure to distal and proximal stressors [14]. Distal stressors include experiences of discrimination and rejection, while proximal stressors are internal processes caused by distal stressors; for example, anxiety, rumination, feeling the need to hide one’s own identity, and holding negative feelings towards one’s own minority group [15–17]. The model was initially developed to explore the detrimental effects of discrimination and stigma on mental health among sexual and gender minoritised individuals [15, 16]. Researchers have also used of the model to explore how discrimination and stigma towards other minoritised groups, such as those who are racially minoritised, also increases their risk of poor mental health [18–21]. By understanding the role of minority stressors in racially minoritised students’ psychological wellbeing, universities and mental health services can implement targeted interventions, and address inequalities and discrimination in HEIs and services.

Racially minoritised students face specific challenges, structural inequalities and Eurocentric curriculums [22]. HEIs are often built on historical colonial ideologies and practices, with racial hierarchies and inequalities embedded within. The presence of statues of and buildings named after colonial figures, such as Cecil Rhodes, have sparked controversy and protests within universities [23]. Lack of diversity among university staff led to the grassroots campaign “*why isn’t my professor Black?”*, which sought to highlight the underrepresentation of racially minoritised academics in HEIs, the systemic barriers racially minoritised scholars face, and institutional racism [24, 25]. Similarly, the “*decolonise the curriculum*” global movement sought to challenge the colonial legacies shaping educational frameworks [26]. These movements demonstrated the ongoing need for HEIs to enhance representation among staff, students, and in the curriculum.

Racially minoritised students may experience covert forms of racism (such as microaggressions) and overt racism both within universities and in wider society. Microaggressions are subtle comments or actions that can marginalise and demean racially minoritised groups or individuals and can lead to isolation and low self-esteem [27]. Carter [28] proposed that such experiences can be sudden, uncontrollable, threatening, and memorable, and can lead to traumatic stress reactions. Smith et al. [29] explored the experiences of Black men in US universities and found that microaggressions can lead to ‘racial battle fatigue’, defined as experiences of hypersensitivity, hypervigilance, fear, and anxiety when entering the predominantly White American academic settings. Understanding the psychological impact of racism is crucial for mental health services which support university students to inform therapeutic interventions and formulations, and challenge systemic discrimination and abuse.

In the UK, the Equality and Human Rights Commission [30] found that 24% of students surveyed had experienced racial harassment and one in 20 university students had left their studies due to racial harassment. Moreover, two thirds of students who had experienced racial harassment did not report it to their university. Universities should work to tackle racism in all forms and support students to report racial harassment to ensure that students feel safe, their wellbeing is supported, and they can complete their studies [31].

### Specific challenges faced by international students

Addressing these issues is particularly pertinent in the US and UK, which have the highest proportion of international students in the world, many of whom may face discrimination and require support for their mental wellbeing [31]. In the UK, approximately 24% of students are international students [32]. International students have become crucial to the financial growth of universities, contributing a fifth of the total income of universities through tuition fees [33]. The presence of international students enriches the academic environment, allowing for cultural exchanges of knowledge and fostering global connections [34].

However, international students face specific challenges when studying abroad; for example, adapting to a new culture and social norms, potential language barriers, loss of social support (such as family and friends back home) and homesickness [13]. International students may experience ‘culture shock’ (such as anxiety and confusion over cultural norms and practices) [34]. Many international students also report feeling isolated from their fellow students [35, 36]. Not only will many international students experience the unique stressors of living and studying abroad, but they may also experience being racially minoritised in their country of study [37]. There was a drastic increase in anti-Asian discrimination following the outbreak of Covid-19, with many Asian international students reporting experiences of racism and abuse [38, 39]. Therefore, there are shared experiences between both home and international students who would identify as from a racially minoritised background within their country of study.

### Help-Seeking for mental health difficulties among students

In 2022, 57% of UK university students reported experiencing a mental health difficulty [6]. Yet Thorley [40] found that almost half of students in the UK did not disclose a mental health condition and that drop-out rates had doubled in the previous 10 years. The most common barriers for accessing support for mental health issues among students are lack of mental health literacy, stigma, lack of awareness of mental health services and perceived inaccessibility of services [41].

### Help-Seeking for mental health among minoritised students

In the UK, rates of disclosure of mental health difficulties by undergraduate university students increased from 6% in 2016/17 to 16% in 2022/23 [42]. However, demographics of students who access services are not representative of the student community [7, 43, 44]. Racially minoritised students, including both home and international students, are less likely to seek help for mental health problems than students from a racial and ethnic majority background; Lipson et al. found that, in the US in 2020/21 55.8% of White students who met criteria for a mental health problem reported seeking treatment for their mental health in the past year, compared to 37.7% of Black students and 35.9% of Latinx students who met criteria for a mental health problem [45]. Racially minoritised students (both domicile and international) experience systemic and personal stressors while at university; for example, experiences of racism, discriminatory policies and lack of diversity in the student and staff populations [36, 37, 46]. Furthermore, racially minoritised students and international students are at an increased risk of experiencing mental health problems while studying [3, 8–13]. For example, Kodish et al. found that Black/African American, Asian American and Latinx students were significantly more likely to screen positive for suicide risk relative to White students [3].

Culture permeates and affects all aspects of mental health experiences [47]. Kleinman et al. [48] used the explanatory model of illness to propose that how individuals understand and experience illness is embedded within a social context. Thus, cultural norms and beliefs impact on how psychological distress is conceptualised, experienced, expressed and responded to [48].

### Rationale

To support the mental health of racially minoritised students, it is important that universities and mental health services understand how these students think about seeking support for their mental health. Previous systematic reviews have explored barriers and facilitators to seeking help for mental health difficulties among young adults and university students; for example, stigma, self-reliance and accessibility of support [49, 50]. Systematic reviews have explored factors associated with help-seeking among people of racially minoritised backgrounds, such as cultural beliefs [51, 52]. However, no review to date has explored attitudes toward seeking help for mental health difficulties among students who are of racially minoritised within the university system. Therefore, we conducted a systematic review to develop a comprehensive synthesis of the existing research, identify common themes across studies and determine possible gaps in research.

### Aim

The primary aim was to explore the perspectives of racially minoritised students on help-seeking for mental health problems by asking the question “what are the attitudes towards seeking help for mental health problems among racially minoritised students?”. A secondary aim of the review was to explore how universities and mental health services can support help-seeking for mental health among racially minoritised students.

## Method

Based on Cochrane guidance, a thematic synthesis approach, as described by Thomas & Harden [53], was used [54]. Attitudes and experiences of mental health difficulties are highly subjective and culture-bound; a thematic synthesis approach allows for integration of diverse perspectives and, by using an inductive approach, it allows researchers to uncover phenomena specific to cultural groups and highlight shared experiences between groups [55]. Moreover, it enables new insights and interpretations that may not be seen in the primary studies [56, 57]. The review was registered to the PROSPERO database (https://www.crd.york.ac.uk/prospero/; ID number: CRD42023485699). Preferred Reporting Items for Systematic Reviews and Meta-Analyses (PRISMA) guidelines for reporting of systematic reviews were followed [58, 59].

### Eligibility criteria

Studies were included if they used direct interviews (such as focus groups and 1:1 interviews), recruited current university students who identified as a racial or ethnic minority within their country of study, included discussion of attitudes toward seeking help for mental health problems, were primary research, applied a qualitative or mixed methods design, and were available in English.

Exclusion criteria were scoping papers or systematic reviews, commentary regarding the subject area, or from books, conference presentations or unpublished dissertations.

### Search strategy

Papers were identified through comprehensive searches. Search terms were linked to the review question, which was devised using the SPIDER tool (Table 1) [60].

**Table 1:** Review question using the SPIDER tool [60]

Element	Term	Criteria
S	Sample	Current university students who identify as from a racial or ethnic minority within their country of study
PI	Phenomenon of Interest	Attitudes toward seeking help for mental health problems
D	Design	Interviews or focus groups
E	Evaluation	Subjective experiences of participants
R	Research type	Qualitative

Search syntax terms were based on key words prevalent in the relevant literature identified through scoping searches; this was discussed with a University Librarian and reviewed with the supervisory team (see Table 2).

**Table 2:** Search syntax

Criteria	Search terms
University student	undergrad* OR postgrad* OR College OR Universit* OR PhD OR doctoral OR student* (Title OR Abstract)
Mental health	“mental health” OR “mental illness*” OR “psych* illness*” OR “psych* problem*” OR depress* OR anxiet* OR *stress (Title OR Abstract)
Help-seeking	“help-seek*” OR “help seek*” OR “seek* help” OR “seek* treatment” OR “seek* support” OR “seek* psych*” OR “psych* help” (Title OR Abstract)
Racially minoritised	Race OR Racial OR Ethnic* OR minority OR Black OR Asian OR divers* OR international (Title OR Abstract)
Qualitative	Qualitative OR Stories OR account* OR views OR interview* OR “focus group*” OR phenomenolog* OR discourse OR thematic OR narrat* OR perspective* OR theme* OR reflecti* (Title OR Abstract)

In December 2023, literature searches were conducted via the following databases: APA PsycINFO, CINAHL, Web of Science and MEDLINE. These databases were chosen as they focused on healthcare disciplines. After titles and abstracts were screened, studies that met the inclusion criteria were retrieved. Full text and hand-searching of references of key studies was completed. Studies were stored on EBSCO, processed using Microsoft Excel and codes synthesised on NVivo (Version 14). Searches were updated in March 2024; 493 possible publications were identified. 15 papers were identified which met the inclusion criteria for the review.

### Reflexive statement

Reflexivity is important to ensure rigour in qualitative research [61]. When completing this review, the primary author considered how their own identity as a White British person could affect the data-analysis; for example, by not having lived experience of being from a racially-minoritised background, the primary author could overlook or minimise important experiences or interpretations. It was therefore important to use a reflexive diary to help the primary author to critically appraise their own interpretations and thoughts and discuss these reflections within the supervisory team. Collaborative reflexivity acknowledges that, to uncover one’s own blind spots, it is necessary to discuss assumptions and decisions with others from a diversity of backgrounds [62]. The research team comprised members of different genders, professional roles, and training backgrounds, allowing for a broad range of perspectives and insights. However, as no member of the team identified as racially minoritised, consultation was sought from an expert by experience who had been an international university student, had experience of seeking help for their mental health problems and was of a racially minoritised background. The primary author’s own experience of being a university student may have influenced the process of creating initial codes and generating analytic themes.

## Results

### Screening and selection

**Fig. 1:**
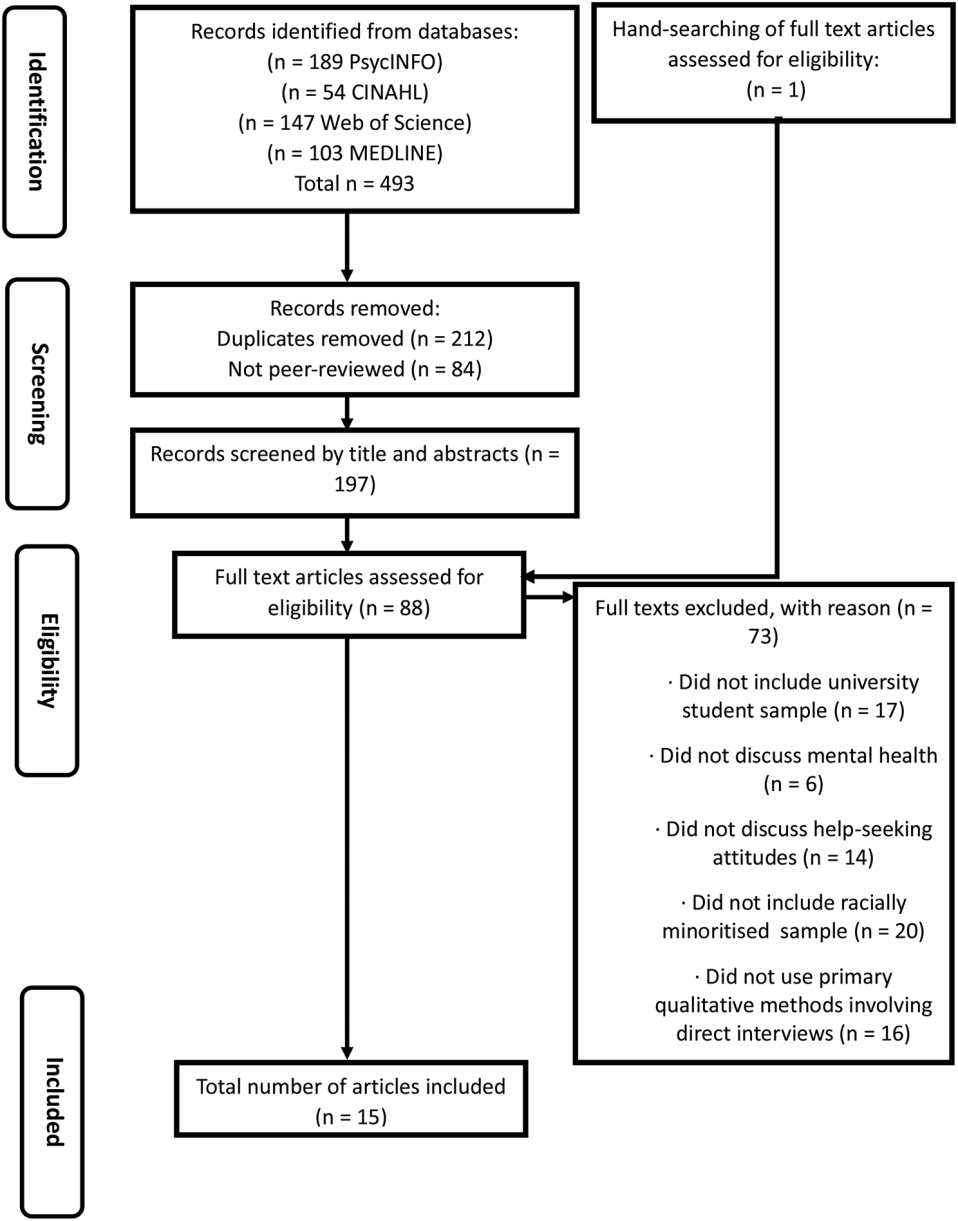
PRISMA diagram

This reviewed followed the methods and results reporting of PRISMA guidelines [58, 59] (Fig. 1). In total, 493 articles were returned in the search (PsycINFO *n* = 189, CINAHL *n* = 54, Web of Science *n* = 147, MEDLINE *n* = 103). Search results were transferred to Endnote (Version 21). 212 duplicates were removed and 84 papers which were not peer-reviewed were removed. One further paper was identified from handsearching. Articles were then transferred to Rayyan (www.rayyan.ai*);* this systematic review tool facilitates the collaboration of the screening process. The title and abstract of these papers were reviewed by the primary author (RH) and 109 were removed through application of the inclusion/exclusion criteria; an independent reviewer (JM) completed the same process on a random sample of 50% of the papers. RH reviewed the full text of 88 articles, and JM completed the same process on a random sample of 10% of the papers. One discrepancy was discussed by the research team, and consensus achieved. Fifteen papers were identified for the final thematic synthesis.

### Quality assessment

The Critical Appraisal Skills Programme (CASP) [63] appraisal tool for qualitative research was used to appraise the papers. This tool uses 10 questions which were scored as follows: yes = 1, can’t tell 0.5, and no = 0. Overall scores of seven and above indicate moderate quality and scores of nine or above indicate high quality [64]. All CASP assessments conducted by RH were independently checked by JM. All discrepancies were discussed between RH and JM to achieve consensus.

The quality of the papers was moderate with overall scores for each paper ranging from 7.5 to 10 (Table 3). Most studies clearly outlined their aims, methodology, design, recruitment, and data collection [65–74]. All studies appropriately outlined their findings and the valuable contribution to clinical practice and research. However, most studies failed to explore researcher positionality and reflexivity.

**Table 3:** Quality assessment outcomes of included studies

Author	Aims	Method-ology	Design	Recruit-ment	Data collection	Researcher relationship	Ethics	Rigour	Findings	Valuable
Arday (2018)	Yes	Yes	Yes	Yes	Yes	Can’t tell	Can’t tell	Yes	Yes	Yes
Attis-Josias (2023)	Yes	Yes	Yes	Yes	Yes	Yes	Yes	Yes	Yes	Yes
Ball et al. (2024)	Yes	Yes	Yes	Yes	Yes	Yes	Yes	Yes	Yes	Yes
Basri et al. (2022)	Yes	Yes	Yes	Yes	Yes	No	Can’t tell	Can’t tell	Yes	Yes
Cogan et al. (2023)	Yes	Yes	Yes	Yes	Yes	No	Can’t tell	Yes	Yes	Yes
Dare et al. (2023)	Yes	Yes	Yes	Yes	Can’t tell	No	Can’t tell	Yes	Can’t tell	Yes
Dong et al. (2022)	Yes	Yes	Can’t tell	Yes	Yes	Can’t tell	Can’t tell	Can’t tell	Yes	Yes
Geegan et al. (2023)	Yes	Yes	Yes	Yes	Yes	No	Can’t tell	Can’t tell	Can’t tell	Yes
Jin & Acharya, (2022)	Yes	Yes	Yes	Yes	Yes	No	Can’t tell	Can’t tell	Yes	Yes
Khatib & Abo-Rass, (2022)	Yes	Yes	Yes	Can’t tell	Yes	No	Yes	Can’t tell	Can’t tell	Yes
Liu et al. (2020)	Yes	Yes	Yes	Can’t tell	Can’t tell	Yes	Can’t tell	Yes	Yes	Yes
Mahmud (2024)	Yes	Yes	Yes	Yes	Yes	No	Yes	Yes	Yes	Yes
Olaniyan (2021)	Yes	Yes	Yes	Can’t tell	Yes	Yes	Yes	Yes	Yes	Yes
Olaniyan & Hayes (2022)	Yes	Yes	Yes	Yes	Yes	Yes	Yes	Yes	Yes	Yes
Sancho & Larkin (2020)	Yes	Yes	Yes	Yes	Yes	No	Yes	Yes	Yes	Yes

### Data extraction and study characteristics

Key information from the studies is in Table 4. The total number of participants across all studies was 314, with a range of six to 48 participants in each study. Seven studies were conducted in the UK [65, 69, 72–76], seven in the US [66–68, 70, 71, 77, 78] and one in Israel [79]. Three studies specifically recruited participants who were international students [68, 69, 71].

**Table 4:** Participant and study characteristics

Author(s)/Year	Aims	Participant Characteristics and study location	Methodology & Analysis	Summary of Outcomes Referencing Attitudes Toward Seeking Help for Mental Health Problems
Arday, (2018)	To explore the barriers to accessing mental health support for and the impact of racial discrimination on students from ethnic minority backgrounds.	32 university students in the UK (F = 18). Asian/Asian British (*n* = 6), Black/Black British (*n* = 14), mixed-heritage (*n* = 9), and Latin-American (*n* = 3). Aged between 18 to 34 years old.	Qualitative design, 1:1 interviewing and thematic analysis.	Themes identified were: how cultural identity and fear of stigmatisation/isolation affect help-seeking; how social networks act as barriers and facilitators to help-seeking; the impact of gender differences on attitudes toward help-seeking; communication barriers and discrimination; power and agency; and language and silencing.
Attis-Josias, (2023)	To add to the theoretical knowledge about the experiences of baccalaureate Black, Indigenous, or persons of colour (BIPOC) nursing students with help-seeking when under stress.	12 nursing university students in the USA (F = 10) who identified as Black (*n* = 3), Hispanic (*n* = 3) and Asian (*n* = 6). Age ranged 21–38 years old.	Qualitative design, 1:1 interviews and thematic analysis.	Themes identified were: lack of diversity among staff and on campus as a barrier to seeking help; and the negative impact of microaggressions and stereotypes on seeking help.
Ball et al., (2024)	To explore how Black students’ understandings of mental health before attending college informed their help-seeking behaviour at college.	48 Black students (F = 36) at US universities who identified as Black/African America (*n* = 28), African (*n* = 12), biracial/multiethnic (*n* = 4), Afro-Caribbean (*n* = 3) and unknown (*n* = 1).	Qualitative design, 1:1 semi-structured interviews and thematic analysis.	Themes identified were: experiences before attending university shape understandings of mental health; difficulties adjusting to university increase likelihood of help-seeking for mental health; negative perceptions of services negatively influence attitudes toward seeking help for mental health; community mental healthcare is often favoured over university-based support.
Basri et al., (2021).	To provide a better understanding of South Asian American college students’ attitudes and behaviours with respect to professional help for psychological and mental health.	14 university students (F = 12) in the USA who identified ethnically as South Asian (Indian *n* = 10, Pakistani *n* = 4). Age range was 18->21.	A qualitative design, semi-structured interviews and thematic analysis.	Themes identified were: how family dynamics affect attitudes toward seeking help for mental health; the impact of the environment outside the home (e.g. the influence of friends and experiences of counselling); the quantity and quality of university resources; ease of/difficulties when accessing help (e.g. affordability); and how cultural dynamics affect attitude toward seeking help for mental health (e.g. stigma and a lack of language/conversation around mental health).
Cogan et al., (2023).	Gaining in-depth insight into the perspectives of Asian International students in their understandings of mental health, experiences of disclosure and help-seeking.	20 Asian International students (F = 10) studying in Scotland, UK who identified ethnically as Chinese (*n* = 9), Indian (*n* = 5), Malaysian (*n* = 2), Saudi Arabian (*n* = 2), Indonesian (*n* = 1) and Iranian (*n* = 1). Mean age = 25.	Inductive, qualitative design with semi-structured 1:1 interviews and thematic analysis.	Themes identified were: how negative beliefs, stigma and fear of judgment negatively impacted on attitudes regarding mental health/help-seeking; the perceived positive impact of cultural adaptation on help-seeking behaviour and negative impact on mental health of acculturation difficulties; how barriers in communication, social disconnection and loneliness negatively impacted on participants’ mental health and inhibited participants from disclosing and help-seeking for mental health.
Dare et al., (2022).	To explore how university students of African, Caribbean and similar ethnic heritage in the UK conceptualise mental health and help-seeking behaviours.	6 University students in the UK (F = 3) who identified as of Black African descent including Nigerian. Age ranged from 22 to 55 years.	Qualitative design and thematic analysis.	Five key themes: perceived meanings and attitudes toward mental health problems; beliefs about the non-existence of mental health problem and its spiritual attributions; family dynamics and the ‘silencing’ of mental health problems; help-seeking for mental health among people of ACE; stigma and discriminatory responses to mental health issues.
Dong et al., (2022).	To understand how the college environment might influence mental health help-seeking in Asian American Undergraduates.	19 undergraduate students (F = 16) in the USA who identified as Chinese (*n* = 11), Vietnamese (*n* = 4), Indian (*n* = 2), Taiwanese (*n* = 1), Filipino (*n* = 1), Cambodian (*n* = 1) and 1 was unreported (note that some participants identified with multiple ethnicities).	Qualitative, semi-structured interviews. Hybrid thematic analysis, using both inductive and deductive codes.	Themes identified were how: the social environment facilitates help-seeking; psychological and physical distance from home facilitates initiation of help-seeking; Asian American-specific experiences can hinder help-seeking.
Geegan et al., (2023).	To explore attitudes, subjective norms and behaviours related to mental health and mental health help seeking among Latinx Gen Z college students.	28 Latinx Gen Z college students (F = 15) in the USA aged 18 to 23 years who identified as Brazilian (*n* = 4), Colombian (*n* = 6), Costa Rican (*n* = 2), Guatemalan (*n* = 3), Honduran (*n* = 4), Mexican (*n* = 5), Nicaraguan (*n* = 1), and Puerto Rican (*n* = 3).	Qualitative methodology using focus groups and inductive thematic analysis.	Four key themes identified were: negative attitudes toward mental health services; Latinx people do not talk about mental health; barriers to accessing help; navigating mental health systems as overwhelming; and structural discrimination.
Jin & Acharya, (2022)	To develop culturally tailored messages for improving mental health and adjustment of (Asian International Students) AISs in the United States.	University students in the USA. Data was only extracted from the focus groups (*n* = 15). Demographics of the focus group not reported but demographics of all participants in the study reported as F = 58, mean age 20.2 (*SD* = 2.13), 46% from China, 15% from India, and 13% from South Korea.	Mixed methods Constant Comparative method from grounded theory using PEN-3 framework.	Themes identified were: need to increase mental health awareness and reducing stigma; to development motivational quotes to promote mental health services; need for available and accessible resources for AISs to improve mental health and adjustment; support of social networks; acculturation; American classroom norms; cultural differences; coping strategies to improve mental health and adjustment; and safety issues for AISs.
Khatib & Abo-Rass, (2022)	To examine mental health literacy among Arab students in Israel based on Jorm’s conceptual framework.	28 participants (F = 16) who were studying in Israel and identified as Arab and were not studying a course related to mental health/ psychology/ psychiatry, aged 21–28.	Qualitative design using 1:1 interviews. Thematic analysis.	Themes identified were: cultural understandings of mental disorders; knowledge and beliefs about risk factors and causes; knowledge and beliefs about self-treatment; knowledge and beliefs about the availability of professional help; attitudes that facilitate recognition and help seeking; stigma of help-seeking and lack of services available in Arabic/in Arab areas; knowledge of how to obtain information about mental health.
Liu et al., (2020)	To understand East Asian international college students’ experiences of mental health counselling in the US.	9 East Asian international university students in the USA (F = 5) from China (*n* = 5) and South Korea (*n* = 4) who had sought mental health counselling within the last 5 years, with a mean age 24.3.	Qualitative design using 1:1 interviews and Interpretive Phenomenological Approach.	Themes identified: perceived influence of social networks on the decision to seek counselling; perceived stigma; expectations of a medical model of counselling; perceived cultural incompetence of counsellor; positive experience of counselling; expectations of the counsellor’s background.
Mahmud, (2024).	To explore the impact of inequality and discrimination on the mental health of Muslim students and accessibility of mental health services at university.	18 undergraduate participants in the UK (F = 8), representative of the ethnic minority demographic (British Muslims from Arab, Asian and African heritage). Age range was 18–25 years old.	Qualitative methodology and thematic analysis.	Themes identified were: the challenges Muslim students face when seeking to maintain their wellbeing due to clashes between religious expectations and secular university environments; how Muslim identity influences how participants think about mental health and help-seeking; and the institutional barriers faced regarding access to mental health support.
Olaniyan, (2021).	To explore the similarities and differences in the lived experiences of racially minoritised students at a Russell Group university and non-Russell Group university.	48 racially minoritised students in the UK (F = 30) who identified as Black British (*n* = 32) and South Asian-British (*n* = 16).	Qualitative paired comparison approach, 1:1 semi-structured interviews and thematic analysis.	Themes identified were: challenges of feeling different to the majority of peers, experiences of mental health difficulties, and seeking help and learning to cope (including lack of cultural competence in services, impact of faith, and self-reliance).
Olaniyan & Hayes, (2022).	Exploring the experiences of and attitudes toward culturally appropriate mental health support for racially minoritised students.	The same sample as above. However, it is not indicated that this is secondary data but seems to have been a pre-planned ancillary study which used different data from the interviews to the data used in the study above.	Qualitative paired comparison approach, 1:1 semi-structured interviews and thematic analysis.	Three themes identified: racially minoritised student preferences towards mental health support regarding cultural appropriateness/ethnic matching in mental health services; culturally focused services; and the need for a reflexive and person specific service.
Sancho & Larkin, (2020).	To understand the barriers and facilitators that Afro-Caribbean undergraduates perceive towards accessing mental health services in the UK.	17 Afro-Caribbean undergraduates in the UK (F = 10) aged 18–25 years.	Qualitative methodology using focus groups, Critical Incident Framework and inductive thematic analysis.	Themes were: psychological barriers/facilitators; sociocultural barriers/facilitators; structural barriers/facilitators; need for targeted interventions and promotion of mental health services for Afro-Caribbean populations; more culturally-sensitive services; greater gender equality; and well-being mentoring schemes with alumni in the universities.

### Data synthesis

Data from the ‘results’ or ‘findings’ section of each paper were extracted to NVivo (Version 14) and analysed using a thematic synthesis approach to explore the attitudes toward seeking help for mental health problems among university students from racial or ethnic minority backgrounds [54]; this included both direct quotes from participants and the interpretations made by the authors of the papers. Free line-by-line coding of each paper was completed, and subsequent ‘descriptive’ themes were identified. These were discussed with the research team and grouped into broader themes; for example, ‘experiences of discrimination’ and ‘family attitudes’. Themes were then compared across the studies to identify similarities, differences and any unique findings. Finally, themes were amended and grouped into overarching categories. This led to the development of the ‘analytical’ themes below.

### Themes

**Fig. 2:**
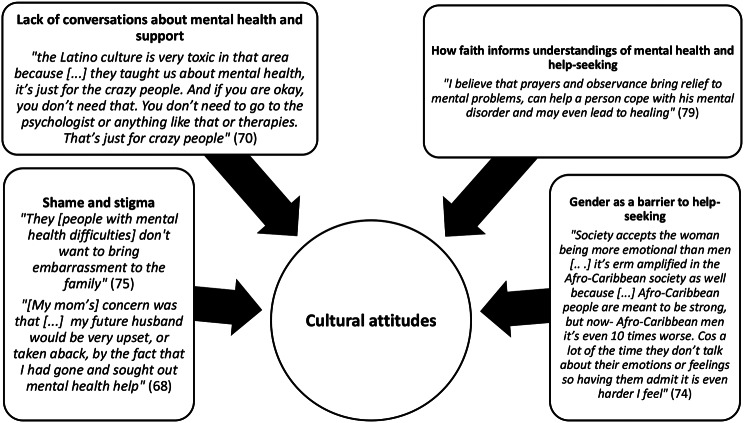
Cultural attitudes: “they taught us about mental health, it’s just for the crazy people” [70]

**Fig. 3:**
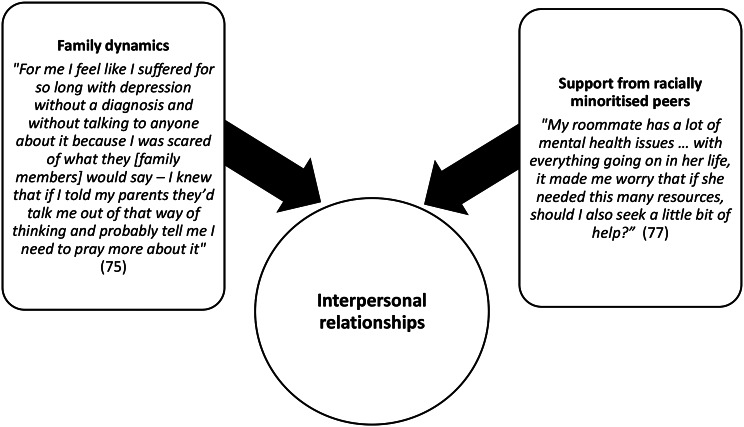
Interpersonal relationships: “Mak[e] sure that the people you associate with don’t look down on mental health” [68]

**Fig. 4:**
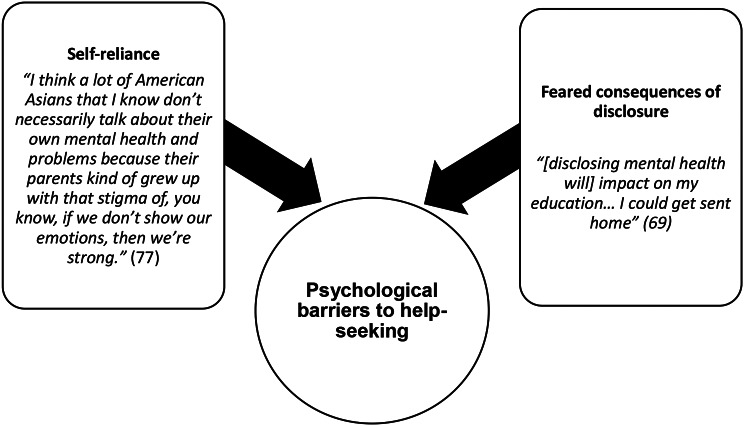
Psychological barriers to help-seeking: “brush it under the rug and carry on” [75]

**Fig. 5:**
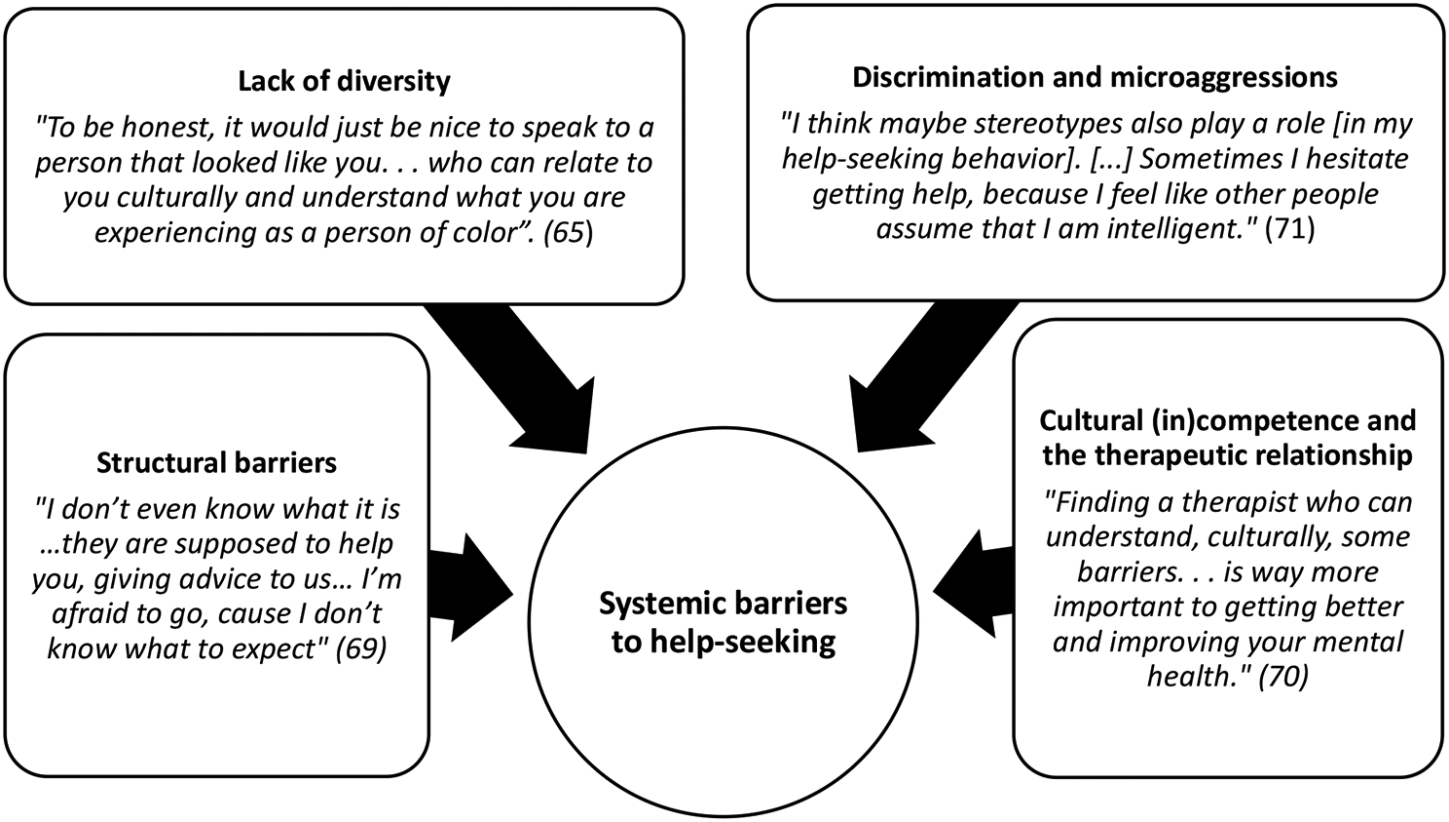
Systemic barriers to help-seeking: “we receive inadequate treatment because of our skin color” [65]

Four themes and 12 subthemes are outlined in Table 5 and with exemplar quotes in Figs. 2, 3, 4 and 5.

**Table 5:** Analytic themes identified

Themes	Arday, J. (2018)	Attis-Josias, M. (2023)	Ball et al., (2024)	Basri et al. (2022)	Cogan et al. (2023)	Dare et al. (2023)	Dong et al. (2022)	Geegan et al. (2023)	Jin & Acharya (2022)	Khatib & Abo-Rass (2022)	Liu et al. (2020)	Mahmud (2024)	Olaniyan (2021)	Olaniyan & Hayes (2022)	Sancho & Larkin (2020)
Cultural attitudes	X		X	X	X	X	X	X	X	X	X	X	X	X	X
Interpersonal relationships	X	X	X	X	X	X	X	X			X	X	X		X
Psychological barriers	X		X		X	X	X	X	X		X		X		X
Systemic barriers	X	X	X	X	X	X	X	X	X	X	X	X	X	X	X

#### Cultural attitudes – “they taught us about mental health, it’s just for the crazy people” [70]

This theme explored culture as a macrosystem, highlighting the broader sociocultural influences that shaped perceptions of mental health and help-seeking. Cultural attitudes were reported as adversely influencing attitudes toward seeking help for mental health problems. Stigmatising attitudes towards mental health and help-seeking within cultural communities negatively impacted on individuals’ views on seeking help for their mental health were common across studies.

##### Cultural attitudes subtheme: shame and stigma

Fear of being stigmatised by cultural communities was reported in all but one study. Individuals feared that their experiences of mental health difficulties would bring shame on their family [65, 68, 69, 71, 72, 74, 75, 78], that it would limit their future opportunities within their cultural community [65, 68] and reported that therapy had negative connotations [76, 78, 79].

*“We Arabs do not look favorably upon mental therapy*,* think that mental therapy is not helpful*,* [and] identify those who seek mental therapy as a weak and crazy person […]. It’s [therefore] difficult to seek treatment and most who do so hide the fact that they’re in therapy”* [79].

*“It’s seen as a characteristic of a problematic family”* [68].

##### Cultural attitudes subtheme: lack of conversations about mental health and support

Silence within cultures around mental and culturally-specific understandings of mental health were reported as barriers to seeking help by participants in several studies [67, 70, 71, 74–78]. Some participants reported their cultural communities only associated ‘mental health’ with severe presentations; for example, someone who is ‘crazy’ or ‘suicidal’ [70, 78, 79]. This limited discussion around more common and milder mental health struggles. This led participants to not recognise that their difficulties were serious enough to warrant support, reinforcing silence, contributing to feelings of isolation and discouraging help-seeking behaviour.

*“Mental health is obviously still not talked about enough”* [70].

*“Growing up […] people weren’t taught about mental health*,* and it inevitably forced my whole household to never discuss mental health*” [67].

##### Cultural attitudes subtheme: how faith informs Understandings of mental health and help-seeking

Participants reported that faith informed how they conceptualised mental health [65, 72–76, 79]. For some participants, mental health problems were seen within their culture as punishment from God; they stated that if they disclosed mental health difficulties to friends or family, they would be told to pray rather than seek formal help [65, 74–76, 79]. This dissuaded some from seeking any help for their mental health difficulties, as they felt they would be judged negatively within their religious community for having a mental health problem or for seeking formal help instead of engaging in religious practices. For others, they reported that they would seek out help for mental health problems from faith leaders and found prayer helpful [69, 72, 73, 76, 79].

*“I think some of those religions and some of the doctrines in the past have given the Black community the impression that mental health is an indication that something is evil or something to be stayed away from.”* [75].

*“If I’m overwhelmed or stressed; they’ll be like it’s because I’m not praying enough*,* or you’ve lost your way on the right path”* [76].

##### Cultural attitudes subtheme: gender as a barrier to help-seeking

Male students faced additional barriers to help-seeking due to cultural beliefs that men should not show their emotions [65, 70, 74, 76, 78]. Moreover, there was a fear that male students from ethnic minority backgrounds, particularly Black students, who sought help for mental health problems would be discriminated against and stereotyped [65, 74].

*“Until they reach a crisis point or rock bottom and*,* as a Black male*,* you are already stigmatized and you are aware that you will be further [stigmatized] upon being diagnosed with this illness*,* which will consequently affect your university studies”.* [65]

“*If the Black male has mental issues you just think he’s aggressive*” [74].

#### Interpersonal relationships: “mak[e] sure that the people you associate with don’t look down on mental health” [68]

This theme explored family and peer relationships as a microsystem, highlighting the impact of family and peer relationships on attitudes toward seeking help for mental health problems. If families had negative attitudes toward seeking help for mental health problems, this increased fear of disclosure and reticence toward seeking help amongst participants.

##### Interpersonal relationships subtheme: family dynamics

Many participants reported that their families did not discuss mental health and would hold negative views regarding seeking help for mental health problems [65, 67, 68, 71, 74, 75, 77, 78]. Some participants reported that their families were supportive of their mental health and help-seeking [67, 68, 74]; however, some stated that, while their families held positive views of help-seeking in general, they would still disapprove if their own family members sought help for mental health problems [68]. Participants stated that having mental health difficulties and seeking help would bring shame on their family; mental health difficulties were associated with dysfunctional families and poor parenting.

*“My parents were aware that I sought counseling at first*,* but they discouraged it […] They were under the impression that if I just tell myself that I won’t have it*,* then I won’t have it.”* [67].

*“It was easier to get help when my parents weren’t there”* [77].

##### Interpersonal relationships subtheme: support from Racially minoritised peers

Participants spoke about how they often felt more able to talk about mental health and seek help if this was modelled by racially minoritised peers [67, 68, 71, 72, 74, 75, 77, 78]. Some international students stated that they did not feel able to speak about mental health or seek help because their fellow international students perpetuated stigmatising beliefs from their home country [69, 78].

*“I feel like people are slowly trying to let people know that it’s okay to not be okay and go seek help.”* [67].

#### Psychological barriers to help-seeking: *“brush it under the rug and carry on”* [75]

This theme highlighted how individually-held psychological constructs were barriers to help-seeking for racially minoritised students; for example, a desire to be self-reliant and fear of the consequence of seeking help.

##### Psychological barriers to help-seeking subtheme: self-reliance

Participants felt the need to keep struggles to themselves and associated help-seeking for mental health with personal weakness [67, 69, 70, 74, 75, 77–79]. Participants spoke about the sacrifices made by their families for them to achieve all that they have, and that that they should therefore be resilient [65, 67, 75]. Some participants spoke about how their families and cultures had struggled for generations with racism, slavery, and civil war; they expressed that they should therefore be able to cope with the struggles of studying at university as these were comparatively less difficult [67].

*“Growing up*,* we operated in a culture of “Suck it up*,*” and type of “You will get over it” type of mentality and vibe. So*,* I feel like structurally*,* when it goes down the line*,* decades and decades*,* families were lynched*,* people weren’t taught about mental health”* [67].

*“Within African families*,* there is an expectation to continuously be resilient as you are reminded of the sacrifices made for you to attend university”.* [65]

##### Psychological barriers to help-seeking subtheme: feared consequences of disclosure

Fear was a psychological barrier to help-seeking for mental health problems, as participants worried that disclosing mental health difficulties could negatively impact on their studies and future employment opportunities [65, 69, 74, 76]. International students worried about lack of confidentiality when disclosing mental health problems and that seeking help would result in them being deported [69, 74]. Students also worried that they would be sectioned, given treatments against their will, and discriminated against because of their racial background [65, 74, 76].

*“My fear is receiving an inappropriate investigation into my psychological state*,* which could affect my studies and potential treatments. As a result*,* between university and my GP. I prefer not to say anything”.* [65]

*“Being Black and mad isn’t a good look out here”* [76].

#### Systemic barriers to help-seeking: “we receive inadequate treatment because of our skin color” [65]

Systemic barriers encompassed the biases within the societies’ policies and institutions that participants faced when seeking help for their mental health problems; for example, experiences of discrimination, lack of diversity on campus and in mental health services, and lack of cultural competence in services. The therapeutic relationship emerged as crucial for addressing these barriers by fostering trust, safety, collaboration, and the feeling of being respected and understood. It was noted that participants felt that this was often lacking in university support and external mental health services.

##### Systemic barriers to help-seeking subtheme: structural barriers

Participants reported that technicalities of health insurance, costs of accessing mental health services, and distance from services prevented them from seeking help [68, 79]. Confusion and uncertainty around how to access support and navigate services was also a key barrier [70, 71, 76]. This was particularly difficult for international students who were unfamiliar with the health systems of their countries of study [69–71, 78].

*“I think for international students*,* everything is a lot harder because we don’t know how anything works. I think that them saying*,* “yeah here we have resources*,*” that’s not helpful. We don’t know how to access the resources*,* even though we know they’re there”* [70].

##### Systemic barriers to help-seeking subtheme: lack of diversity

Participants noted a lack of racial and ethnic diversity on campus and within mental health services [65, 66, 72–74, 76, 78]. They perceived that university staff and mental health professionals (MHPs) would not understand their experience, which prevented them from seeking help.

“*You feel weird being a Muslim in a place where you do not see any Muslim staff*,* you don’t know who to approach and how to do it and if you’re going through anxiety and stress*,* this can seem like a bigger mountain to climb.”* [72].

*“There aren’t enough minority ethnic people within the mental health system”* [75].

##### Systemic barriers to help-seeking subtheme: discrimination and microaggressions

Microaggressions and perpetuation of stereotypes by university staff and by MHPs had a negative impact on students’ mental health and attitudes toward seeking help [65, 66, 76, 78]; for example, Asian students reported that staff perpetuated the “model minority” stereotype, which led them to not want to ask for help.

*“When I told [my therapist] I am from South Korea*,*. She said “Oh*,* your English is really good”; I don’t know what she meant. I felt negative. Maybe she does not really have much experience with Asians.”* [78].

*“We’ll be treated lesser than our White counterpart”* [76].

##### Systemic barriers to help-seeking subtheme: cultural (in)competence and the therapeutic relationship

A lack of diversity within universities and mental health services coupled with discrimination and microaggressions led participants to feeling that institutions were not culturally competent [65, 66, 68, 70, 72–76, 78]. Students felt that professors and MHPs did not understand their cultural background, minimised experiences of racism, isolation, and culture shock, and were not willing to discuss race and religion [65, 67, 70, 72–74]. This negatively impacted on the therapeutic relationship and made students feel unwilling to seek help for their mental health problems. Some participants reported that they felt these barriers could be overcome if MHPs were open to learning about a culture, respectful and empathetic [73, 76, 78].

*“If you can’t understand us as people*,* then you’re not going to understand our mind”* [67].

*“I would prefer to have a psychological doctor who can speak some Chinese and was raised in Chinese culture……[but] barriers of gender*,* race*,* and language can be overcome. It will be fine if the doctor can understand me.”* [78].

## Discussion

The review identified that cultural attitudes encompassing stigma, understandings of mental health, faith and gender norms significantly influence attitudes toward help-seeking. Family attitudes towards seeking help for mental health problems can also exacerbate or alleviate students’ mental health. Supportive peer relationships and modelling facilitate positive attitudes towards help-seeking. Participants demonstrated how individually-held psychological constructs can act as barriers to seeking help, including the need to be self-reliant and fearing the consequences of disclosing mental health difficulties. Additionally, systemic barriers, such as structural barriers, a lack of diversity on campus and in mental health services, experiences of discrimination and microaggressions, and perceived cultural incompetence of mental health professionals, pose critical challenges for racially minoritised students’ mental health help-seeking. Multifaceted and nuanced influences on mental health help-seeking for racially minoritised students were apparent and highlighted the need for strategies which address the barriers and foster an inclusive and supportive environment for racially minoritised students.

Limited mental health literacy, stigma, lack of awareness of mental health services and perceived inaccessibility of services affect attitudes towards seeking help for mental health problems amongst the general university student population [41, 49, 50]. This review also found that these factors influenced the attitudes of racially minoritised students, but in addition found that factors were compounded by the experiences of being minoritised and cultural beliefs.

Male students, regardless of racial or ethnic background, are less likely than female students to seek help for their mental health problems [80–82]. However, racially minoritised men are less likely to access mental health support in primary care and are more likely to access mental health services through the criminal justice system than White men [83]. This study found that male students faced additional barriers to help-seeking, and thus is comparable to studies with non-racially minoritised populations. However, racially minoritised men faced additional barriers, such as fear of being sectioned or treated inappropriately based on racial biases. Therefore, while gender disparity in help-seeking is evidence across racially populations, racial discrimination and stereotypes further compound negative attitudes towards seeking help for mental health problems amongst racially minoritised men.

Lack of awareness of mental health support is a commonly reported barrier to help-seeking among general student populations [41, 49, 50, 84]. However, racially-minoritised (and particularly international students) reported fearing that disclosure of mental health difficulties could result in them being forced to leave the university. This demonstrates a difference between attitudes towards help-seeking among racially minoritised groups and general student populations. Greater promotion to racially minoritised and international students of mental health services, how to access them, and services’ responsibilities to ensure confidentiality is necessary to build awareness and trust.

Racially minoritised students often feel reluctant to seek help due to the belief that self-reliance is indicative of personal strength, while help-seeking is associated with weakness [51]. Notions of self-reliance are also a commonly reported barrier to seeking-help for mental health problems [49, 50]. However, specific to racially minoritised students, reluctance to seek help appeared due to beliefs that they should be able to cope independently, particularly given the challenges their families and communities had faced historically. Wellbeing resources provided to students could challenge notions of self-reliance to reduce this barrier to help-seeking. MHPs working with racially minoritised students should be mindful of how self-reliance may impact on psychological wellbeing; for example, how seeking help may negatively impact on self-esteem if an individual believes that they should be self-reliant and that seeking help is a weakness. Psychotherapeutic approaches which target unhelpful core beliefs while also recognising and respecting individual strengths and cultural contexts, such as culturally adapted cognitive behavioural therapy, could be utilised to modify these cognitive patterns [85].

Social support is associated with good mental and physical health [86, 87]. However, many racially minoritised students felt unable to discuss mental health or help-seeking with their family due to fear of their response; students described feeling pressured by their families to excel academically and feared that seeking help for their mental health problems would be perceived as failure and would bring shame to their family. Public stigma is often experienced by families of those with mental health difficulties and has been shown to be associated with greater psychological distress and less perceived closeness between family members [88–90]. Therefore, interventions targeting stigma and mental health literacy at the levels of communities and families could reduce public stigma, increase levels of social support, and reduce barriers to help-seeking for mental health problems.

Peer support can facilitate help-seeking for mental health difficulties, particularly within the student population, where loneliness and isolation are prevalent [91–93]. Social learning theory proposes that people learn through observing the actions of others [94]. This review found that, similar to findings in non-racially minoritised student populations, peer support is crucial in encouraging help-seeking behaviour [91–93]. However, a key difference is that racially minoritised students felt that talking to fellow racially minoritised peers about mental health was more helpful than accessing other sources of support. They were also more likely to seek help if this had been modelled by racially-minoritised peers. Mental health first aid training for students has been shown to be effective in improving self-awareness of mental health and when to seek help, as well as increasing students’ confidence in their abilities to help peers who are experiencing a mental health difficulty [95, 96]. Developing culturally-sensitive mental health first aid training and promoting this within diverse student populations could enhance peer support systems and promote professional help-seeking [97].

The minority stress model can be applied to the experiences of racially minoritised students to demonstrate how stressors (such as microaggressions, discrimination and self-stigma) contribute towards poor mental health [18]. Indeed, the university environment can have a significant detrimental effect on mental health and help-seeking attitudes for racially minoritised students [27, 76, 98]. This review corroborated previous findings that overt and covert experiences of racism (such as microaggressions) impacted negatively on the mental health of racially minoritised students [27–29]. Moreover, experiences of microaggressions prevented students from seeking help for their mental health difficulties. This presents a concerning picture that racially minoritised students are both at increased risk of mental health problems because of these experiences and feel unable to seek appropriate support for their mental health because of microaggressions and discrimination.

A lack of MHPs from racially minoritised backgrounds is also a barrier to help-seeking. The review found that many participants would favour an ethnically-matched MHP to work with, and that all participants felt that MHPs and universities needed to develop cultural competence. Increased training and recruitment of racially minoritised MHPs and additional training to develop cultural competence among MHPs is essential to culturally representative and sensitive workforces. This review found that language was a key barrier to accessing support. Culturally-sensitive services must therefore be able to meet the linguistic needs of students by ensuring that staff are representative of the communities they serve and that translator services are available.

### Strengths and limitations

A notable strength of this review was that the robust search strategy, which included searches from multiple databases and handsearching, produced a homogenous sample of high quality studies with robust methodologies whose aims related to the review question. Moreover, it focused on a particular phenomenon; while attitudes toward seeking help for mental health problems have been researched and reviewed for students in general, reviews have not explored experiences and attitudes of racially minoritised students. The research evidence discussed above shows that racially minoritised students are unlikely to disclose and seek help for their mental health problems [12, 42, 83, 84]; the current review contributes to understandings of why this may be the case.

Despite no limit on year of study publication, all studies included were conducted within the past six years, with six studies being published in the last year; this implies that this is an emergent area of research. Furthermore, as universities are seeking to be more inclusive and diverse, this synthesis of the current evidence is particularly timely and relevant.

A key limitation of this review was that ‘racially minoritised’ is an umbrella term for multiple identities, within which there is heterogeneity of experiences. Therefore, there is a risk of overgeneralising and overlooking important, culturally-specific differences. Furthermore, studies in the UK, the US and Israel were included; while there were shared experiences and themes identified, there will be key differences in healthcare and university systems in different countries. Further research could explore differences and similarities in specific countries and racially minoritised groups. Furthermore, a feature of qualitative research that could be perceived as a limitation is that qualitative research is not intended to be generalisable; however, it does provide valuable insight into the experiences of participants and helps to identify future research areas. Finally, 10 out of the 15 papers included did not include adequate reflexivity and researcher positionality. It is important that researchers engage in critical reflection on how their personal characteristics, experiences and beliefs can shape the research and its outcomes.

### Implications and future research

**Fig. 6:**
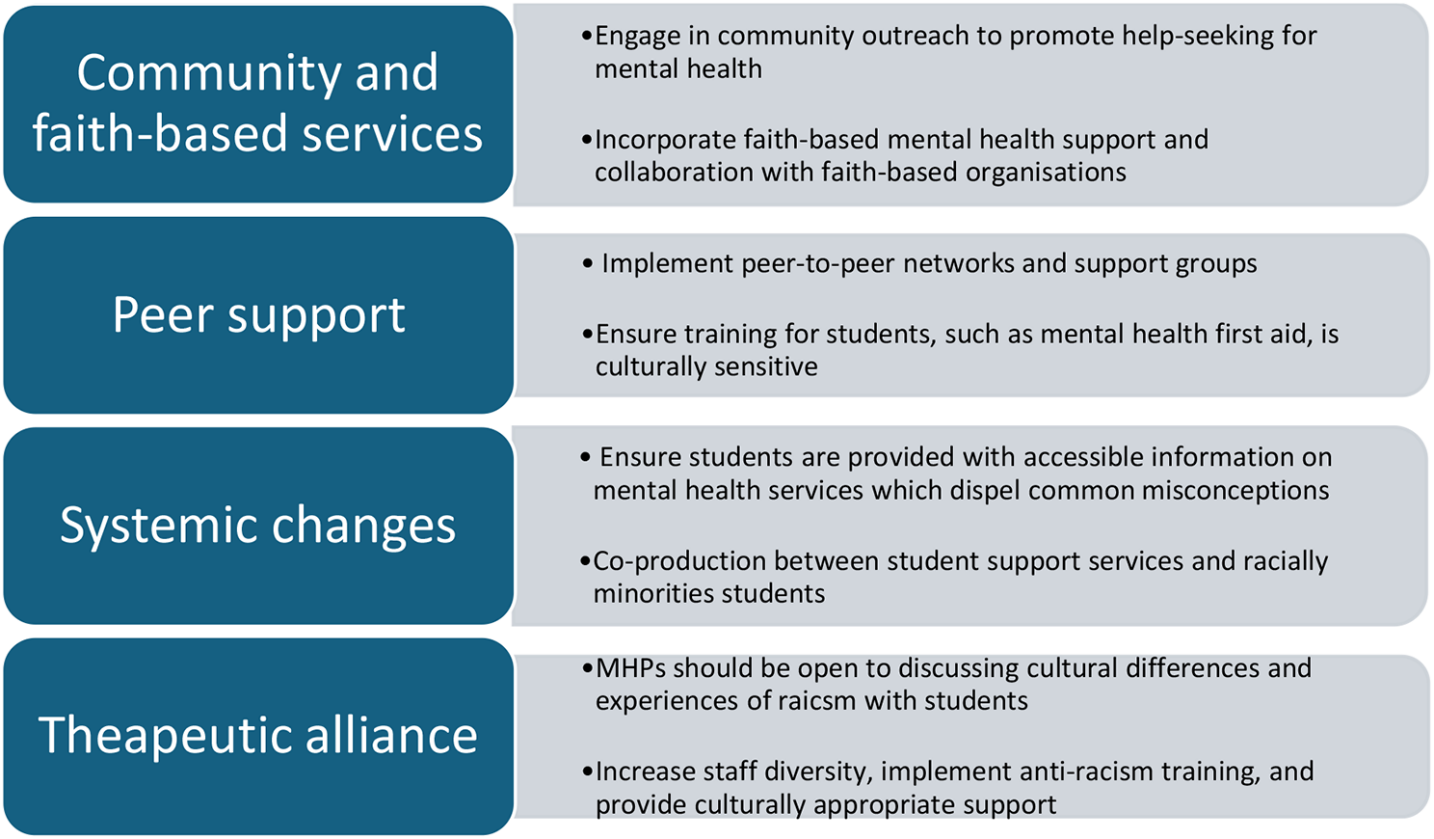
Recommendations for Universities and Mental Health Services

Understanding the barriers faced by racially minoritised students is crucial for universities and mental health services to provide support. Recommendations for universities and mental health services are detailed in Fig. 6.

This review has shown the impact on individuals’ mental health and attitudes toward help-seeking of cultural understandings and stigma of mental health problems, gender norms and pressures to be self-reliant. MHPs could engage in community outreach and provide tailored resources for universities which focus on psychoeducation. MHPs working in student services should facilitate collaboration between mental health services, faith-based organisations and racially minoritised students and could help to develop culturally-sensitive resources and support.

This review has also demonstrated how social support can impact on attitudes toward seeking support for mental health problems among racially minoritised students. Peer support was demonstrated to be a key facilitator to help-seeking. Universities could improve psychological wellbeing and attitudes toward seeking help for mental health problems among racially minoritised students through peer-to-peer networks and support groups, providing training to these groups, such as culturally sensitive mental health first aid.

Many students reported feeling unsure what services were available to them, how to navigate the systems and felt concerned about the consequences of seeking help. Greater information on the services available and on confidentiality and its limits should be communicated to students to alleviate anxiety.

Systemic change within universities and mental health services is necessary to increase the accessibility of mental health support for racially minoritised students. MHPs can utilise their positions within services and their research skills to advocate for policy change and initiatives within universities and mental health services that will promote equality, diversity and inclusion. It is important that universities and mental health services increase staff diversity and are representative of the populations that they serve, engage in anti-racism training (including increasing awareness of unconscious biases and the unfair treatment of people of racially minoritised backgrounds), provide culturally-sensitive and appropriate support, and provide greater availability and promotion of translator services. This is critical to improve care for racially minoritised students and facilitate more positive attitudes toward seeking help for mental health difficulties.

Several policies in the UK have highlighted the pervasive mental health inequalities experienced by racially minoritised groups, in particular Black men [99, 100]. The Patient and Carer Race Equality Framework (PCREF) seeks to embed anti-racist practice and policies in NHS mental health services to advance mental health equalities [101]. Underpinning this is the NHS Constitution, which details that it is every professional’s duty to ensure the human rights of patients are upheld and equality is promoted [102]. Uptake of the PCREF in student services could facilitate more co-production with racially minoritised experts by experience of policies, guidance, and services, and tackle structural discrimination and inequality. Greater integration of research on racial discrimination and gender-related biases should be incorporated into training of MHPs and university staff who support students.

The therapeutic alliance is central to building culturally sensitive and appropriate services. The therapeutic alliance refers to the collaborative relationship between a professional and client. Bordin [103] argued that the therapeutic alliance consisted of three elements: shared treatment goals, agreement on tasks, and the development of a strong therapeutic relationship. The therapeutic alliance has been consistently linked to positive treatment outcomes [104–106]. This review found that participants felt cultural barriers could be overcome if MHPs were open, respectful, and able to talk about differences and experiences of racism; this could strengthen the therapeutic relationship. Enactment of racial microaggressions and discrimination by MHPs will have a detrimental effect on the therapeutic relationship, further exacerbated by the power differentials between MHPs and clients [107]. MHPs working therapeutically with racially minoritised students should be open to discussing differences in identity and how this could affect the therapeutic relationship, as well as the psychological impact of experiences of discrimination and racism. Doing so will facilitate a respectful, trusting, and collaborative therapeutic alliance.

Future research should build upon the limitations of this study and seek to explore experiences of and attitudes toward help-seeking among specific racially minoritised student populations. Future research should consider intersectionality; while this review has focused on race and ethnicity, it has demonstrated that there is overlap with faith, language, gender, and nationality. Moreover, exploration of the impact of racial and ethnic differences between MHPs and clients on the therapeutic alliance is integral to improving care and therapeutic outcomes. For universities and mental health services to meet the needs of underserved and at-risk groups, there needs to be more understanding of the complex and multifaceted aspects of identity that affect mental health and attitudes toward and experiences of seeking help.

## Conclusion

Culture, identity and social inequalities play crucial roles in shaping attitudes towards seeking help for mental health problems among racially minoritised students. These attitudes are influenced by psychological constructs (such as beliefs and fears), interpersonal relationships, cultural norms, and broader structural barriers and structural inequalities. To enhance the provision of mental health support for racially minoritised students, systematic changes are necessary in universities and mental health services.

## Data Availability

The datasets used/or analysed during the current study are available from the corresponding author on request.

## Abbreviations

HEIs: Higher Education Institutes
PRISMA: Preferred Reporting Items for Systematic reviews and Meta-Analyses
CASP: Critical Appraisal Skills Programme
PCREF: Patient and Carer Race Equality Framework
BPS: British Psychological Society
